# The drug drought in maternal health: an ongoing predicament

**DOI:** 10.1016/S2214-109X(24)00144-X

**Published:** 2024-06-12

**Authors:** Anne Ammerdorffer, Annie R A McDougall, Andrew Tuttle, Sara Rushwan, Lester Chinery, Joshua P Vogel, Maya Goldstein, A Metin Gülmezoglu

**Affiliations:** a Concept Foundation, Geneva, Switzerland; b Maternal, Child and Adolescent Health Program, Burnet Institute, Melbourne, VIC, Australia; c Policy Cures Research, Sydney, NSW, Australia

## Abstract

We developed a comprehensive database of medicines that are used or are being investigated for pre-eclampsia or eclampsia, preterm birth or labour, postpartum haemorrhage, intrauterine growth restriction, and fetal distress and that were in active development between 2000 and 2021. A total of 444 candidates were identified: approximately half of candidates were in active development, two-thirds had been repurposed after initially being used for another condition, and just under half were in preclinical studies. Only 64 candidates were in active late-stage (phase 3) development as of Oct 25, 2021, and given the slow pace of biomedical development, it could take years before any of these products eventually make it to market. A lack of innovation for maternal health medicines persists, and the market continues to fail pregnant individuals. There is a need for collective action from all relevant stakeholders to accelerate investment in the development of new or improved medicines for pregnancy-related conditions.

## Introduction

Despite improvements in maternal health outcomes over the past two decades, approximately 287 000 women still died from pregnancy-related causes in 2020, of which most lived in low-income and middle-income countries (LMICs).^1^ The prioritisation of maternal health within the Sustainable Development Goals—and the Millennium Development Goals before them—has focused largely on improving access to and quality of existing interventions and maternity services. Although this focus has resulted in substantial public health benefits, including a 34·3% drop in the global maternal mortality ratio between 2000 and 2020,^1^ there has been little attention or credence given to biomedical research and development (R&D) for new medicines for pregnancy-specific conditions.

In 2008, Fisk and Atun^2^ published an important analysis of the drug development pipeline for maternal health medicines, which placed a spotlight on a chronic and chronically overlooked problem: how few medicines were in the R&D pipeline for obstetric conditions. They reported that, over a two decade period, only 67 drugs had been investigated for a maternal health indication (the majority of which were for preterm labour or preterm birth), and just 17 drugs were under active development for a maternal health indication at the time of analysis (panel 1).^2^ Only one new drug, atosiban for preterm labour, was available to patients and only in high-income country settings. Although making comparisons between the pregnancy drug market and other drug markets is complicated (given the differences in market sizes and disease prevalence), it is striking how many more candidates are under development for much rarer conditions than those associated with pregnancy. Fisk and Atun reported double the number of pipeline candidates (34 candidates in total) for the rare neurodegenerative condition amyotrophic lateral sclerosis compared with candidates for pregnancy-specific conditions.^2^ A separate analysis found 440 candidates for paediatric cancers were trialled over a 15 year period (2007–22), of which 92 products were marketed.^5^

Panel 1 **What happened to the 17 drugs under active development for maternal health indications identified by Fisk and Atun in 2008?**

In 2008, Fisk and Atun^2^ identified 17 active candidates: ten for preterm labour or birth (labour inhibition); two for pre-eclampsia or eclampsia; one for pre-eclampsia or eclampsia and labour induction; two for labour induction; and two for miscarriage. Only preterm labour or birth and pre-eclampsia or eclampsia are pregnancy-specific conditions that are part of our scope, and therefore only the 13 candidates with these indications were available for comparison.

Of these 13 candidates, only two were still in active development at the time of our study: injectable hydroxyprogesterone caproate (distributed under the name Makena, previously Gestiva) and vaginal progesterone. Injectable hydroxyprogesterone caproate was approved by the US Food and Drug Administration (FDA) in 2011 for prevention of preterm labour in at-risk individuals, and vaginal progesterone is widely used off label for prevention of preterm labour in people with a short cervix, with recent evidence of ongoing clinical investigations for both products. However, both candidates are also affected by controversy, with major long-term clinical trials demonstrating questionable efficacy, effectively culminating in the FDA request for Makena's withdrawal in 2020, and then the official withdrawal from the market in 2023.^3^

The remaining 11 candidates were inactive at the time of our study. Among these candidates were the novel oxytocin receptor antagonist GSK221149A (also known as retosiban) and all three pre-eclampsia or eclampsia candidates, referred to by Fisk and Atun^2^ as relaxin, Digibind, and digoxin antibody (the last two candidates are considered as one drug candidate in our pipeline). The digoxin antibody (AMAG Pharmaceuticals; Waltham, MA, USA) has been granted orphan drug status for treatment of severe pre-eclampsia or eclampsia by the FDA in 2012,^4^ though no active development has been detected. The phase 2 research into nolasiban (NCT02326142), which probably superseded or was a continuation of AS-602305 reported in Fisk and Atun's paper, was terminated due to enrolment difficulties.

Reactions to Fisk and Atun's analysis began appearing from 2015, with a UK Royal College of Obstetricians and Gynaecologists statement,^6^ opinion papers,^7^ studies on the ethics of pregnant women's inclusion in clinical research,^8^ and special initiatives by regulatory agencies^9^ and national research institutes.^10^ These papers highlighted the particular challenges in evaluating new medicines during pregnancy, the over-reliance on repurposed and off-label medicines for pregnancy-related complications, how existing market mechanisms have failed pregnant people, and how new investments are sorely needed. Little access to new, purposively developed medicines is even more concerning when considering the perspective of individuals living in resource-limited settings, where the burden of preventable maternal mortality and severe morbidity is greatest.^1^ Underinvestment in medicine and vaccine R&D for pregnancy-related conditions has since been echoed in several publications.^6^, ^7^ For example, early in the COVID-19 pandemic, it became clear that pregnant women were being systematically excluded from COVID-19 vaccine and treatment trials, generating renewed calls for reform.^10^, ^11^

In 2021, we launched the Accelerating Innovation for Mothers (AIM) project, which aims to develop pathways for accelerating development and introduction of innovative products for pregnancy-specific conditions. As part of AIM, we developed a comprehensive database of medicines that have been used or investigated at any point, and that were at any stage of development, for preterm labour or birth, pre-eclampsia or eclampsia, intrauterine growth restriction, postpartum haemorrhage, and intrapartum fetal distress over a 20 year period (2000–21). These five pregnancy-specific conditions are responsible for most of the direct maternal deaths and severe maternal morbidity occurring globally.^12^ Despite this burden, there have not been any new medical innovations for these conditions that reflect the current knowledge of disease evolution and progression processes. In addition, we investigated the current market challenges and potential solutions for the development and introduction of medicines for pregnancy-specific conditions through literature reviews and stakeholder consultation,^13^ and by developing target product profiles (a document describing the minimum and preferred, or optimal, characteristics of a target product, aimed at a particular disease) to better direct maternal health medicine R&D.^14^, ^15^ In this Health Policy, we present the methods and main findings of the AIM database of maternal health medicines and discuss the ongoing market failures that need to be addressed. Although our current research focuses on the five high-priority, pregnancy-specific conditions discussed above, we acknowledge the impact of a wide range of other pregnancy-specific conditions, which are also chronically under-represented in clinical research and funding. In addition, non-communicable and communicable diseases can also affect pregnant people, in some instances causing worse outcomes than for people who are not pregnant.

## Methods

We aimed to create a comprehensive database profiling all medicines (drugs, biologics, and dietary supplements) investigated between 2000 and 2021 for preterm labour or birth, pre-eclampsia or eclampsia, intrauterine growth restriction, postpartum haemorrhage, and intrapartum fetal distress.^16^, ^17^ The comprehensive methodology for the development of the database can be found online.^16^ The interactive database, which includes the data used in this paper, is freely available.^17^ For this study, the maternal health pipeline database version from Oct 25, 2021, was used. The five conditions included in our study are not the only pregnancy-specific conditions, but within the limits of the duration and funding for the study, we prioritised these five conditions because they are major drivers of maternal and perinatal morbidity and mortality globally.

### Eligibility criteria

For inclusion in the database, the medicines needed to meet the following criteria (see panel 2 for additional information on types of data collected). First, they needed to be small molecules (drugs), biological products (eg, growth factors, immune modulators, monoclonal antibodies, and products derived from human blood or plasma), or dietary supplements. Candidates could be entirely new entities; existing, repurposed, or label extensions; or new formulations or doses of existing or registered medicines. Second, they had to have an indication or multiple indications related to the project's five identified pregnancy-specific conditions: preterm labour or birth, pre-eclampsia or eclampsia, intrauterine growth restriction, postpartum haemorrhage, or fetal distress. Third, they had to be in active development, or have been in development at some point between January, 2000, and December, 2020 (public announcements and updates on relevant candidates made between January, 2021, and May, 2021, were also captured). And fourth, they had to be either investigated for clinical use or used currently in clinical treatment, or both, specifically for the five identified conditions.

Panel 2 **Pipeline data fields and related methodological refinements**

The data fields included in our pipeline evaluation were: candidate name, alternative or previous candidate names, pregnancy-specific condition (primary), indication, archetype, product type, WHO Anatomical Therapeutic Chemical code, medical subject headings, pharmacological subgroup, route of administration, target, mode of action, clinical use status, current research and development (R&D) stage (for this pregnancy condition), highest R&D stage (for any condition), development status, inactive development type (as appropriate), key features or challenges, most recent update, US Food and Drug Administration (FDA) pregnancy labelling and pregnancy risk summary, preclinical results status, preclinical results type (as appropriate), preclinical results source (as appropriate), investigated for other indications (yes or no), other indications (as appropriate), developer, patent, Chemical Abstracts Service number, and chemical name.

For each field, a definition, data input description, and sample data type classification (numeric, free-text, defined list, etc) was developed, as well as guidance notes when relevant, to ensure standardised data entry across researchers and enumerators.^17^

Specific and important definitions were as follows:

• Archetype: repurposed candidates were defined as any candidate previously or currently marketed for any other condition. New chemical or biological entities (NCEs) are candidates that are not already marketed for any condition, unless a now marketed product was developed as a NCE for an inscope pregnancy-related condition (eg, atosiban is a NCE when it was developed and is now marketed for preterm labour, whereas under fetal distress, atosiban is a repurposed drug, as it is already marketed for another condition [preterm labour]). We also considered candidates with new formulations or different routes of administration of existing medicines (inhalable oxytocin, heat-stable carbetocin, etc) to be NCEs, unless they were already marketed for some other use.
• Clinical use status: if verified in the literature, drug databases, FDA or European Medicines Agency sites, or by one or more clinical practitioners or experts that a candidate was approved and marketed for clinical use or is advised for or frequently used off label for that condition in clinical practice, candidates were marked as such. Off-label drug use refers to the practice of prescribing a drug for a different purpose than what the FDA or other stringent regulatory authority approved. This practice is called off-label use because the drug is being used in a way not described on its package insert. Candidates that were approved and then withdrawn from the market were marked as withdrawn. All other candidates that were not approved, withdrawn, or used off label were marked as investigational.
• Current R&D stage: some candidates were in clinical development with no R&D stage specified in linked clinical trials, or the stage was stated as not applicable or unknown. Other candidates had a variety of R&D stages listed among various clinical trials, including those marked as phase 4, even for candidates not yet marketed for that condition. To allocate a single, appropriate R&D stage to each candidate, we reviewed linked clinical trials against accepted definitions of each R&D stage (preclinical through to phase 4), and assigned a phase based on trial descriptions.
• Development status (active *vs* inactive): due to the often proprietary nature of R&D, information on the current development status of some candidates was not available in the public domain. To avoid labelling many candidates with a development status of unknown, it was agreed that active candidates would be defined as any candidate with evidence of R&D within the last 3 years (since 2019). If no updates were made available on a candidate in the previous 3 years, or there was clear evidence of their discontinuation since then, they were classified as inactive.

We excluded the following: devices, diagnostics, or other non-medicine related biomedical products; dietary supplements without dosed formulations (ie, food-based interventions such as beetroot juice or chocolate); and candidates directed at health conditions related to the pregnancy-specific conditions, but not at the five conditions themselves (eg, a medicine targeting cardiac disease in women who have had pre-eclampsia).

During pipeline development, we identified some contentious areas related to pipeline scope. Pragmatic decisions were taken to limit scope creep, particularly for medicines that are used regularly in pregnancy for other reasons, and to ensure the pipeline was focused on candidates specific to these five conditions.

Specifically, despite the only cure for pre-eclampsia being delivery, labour induction drugs were not included for this condition. Such drugs are indicated for labour induction for any reason, and not for specific use in managing pre-eclampsia. Antibiotics (azithromycin, erythromycin, clindamycin, amoxicillin, etc) investigated in or used for preterm labour or premature rupture of membranes, or as a prophylactic in pregnant individuals with intact membranes, were excluded based on their broad applicability well beyond the pregnancy-specific conditions in question, the large number of potential candidates (with potential to skew the dataset), and their downstream position in treating each condition's pathology. Several studies report on the development pipeline of antibacterial agents.^18^, ^19^ Antihypertensives were included when they had been tested in or used directly for pre-eclampsia—despite their broad applicability beyond pregnancy—given their specific and critical use to prevent and treat this condition. Antenatal agents aimed at reducing the consequences of preterm labour or birth (eg, corticosteroids for fetal lung maturation) were included since they have indications and evidence of investigation specific to preterm labour. Agents that inhibit uterine infection or inflammation (acetylcysteine, indometacin, aspirin, progesterone, pravastatin, etc), or maintain vaginal flora or pH, were included due to their specificity to the inflammatory pathways identified as precursors to a number of the pregnancy-specific conditions,^20^, ^21^ and their upstream position in treating the condition's pathology. Haemostatic agents for treatment of general haemorrhage (prothrombin complex concentrate, fresh frozen plasma, etc) were included only if postpartum haemorrhage was specifically indicated for its use or was investigated as an outcome. Candidates used only for experimental purposes (eg, as an experimental aid to elicit smooth muscle contraction or relaxation) were only included if the research was specifically geared towards one or more of the five pregnancy-specific conditions.

### Sources

We undertook four steps to develop the database of candidate profiles to fill 30 predefined data fields (when data were available and verifiable; panel 2).

#### Step 1: initial candidate identification

Step 1 included the initial candidate identification through searches in pharmaceutical databases (AdisInsight, and Citeline's Pharmaprojects), clinical trial registries (WHO International Clinical Trials Registry Platform [ICTRP], which provides information on trials listed on ClinicalTrials.gov and all other relevant national and international clinical trial databases), peer-reviewed literature (PubMed), and grant databases from top funders of global maternal health (the US National Institutes of Health's RePORTER, the European Commission's CORDIS, and the Bill & Melinda Gates Foundation grant database). Search terms used for each database were developed iteratively and are specified in the methods of the report published online.^16^

#### Step 2: linking preclinical and clinical development data

For candidates in clinical development, we collected relevant clinical trial data through two main sources. First, primary candidate identification through AdisInsight (step 1) also provided linked clinical trials. Second, we datamined the datasets retrieved from the WHO ICTRP. Both were scoped for relevance, and data was manually uploaded to the clinical trial entries in our database.

#### Step 3: completing candidate profiles

Steps 1 and 2 captured most of the information needed to complete candidate profiles. Step 3 was performed to complete the candidate profiles and to add additional contextual information to each individual product. We used PubMed searches of the candidate names and reviewed relevant literature retrieved (including citations already sourced in step 1) to verify and cross-reference information as needed. For candidates that are no longer in development or have not shown any recent progression, additional information was searched via relevant regulatory websites. Information for other database fields were sourced from a number of reliable online sources, including DRUGBANK Online, PubChem, and the US National Library of Medicine's Medical Subject Headings portal.

#### Step 4: external validation and sense checking

The database was validated and sense-checked through rigorous review by at least two individuals from the research team, followed by a macro review from the broader project team. The complete database was also reviewed by two external independent specialists working on drug development for one or more of the pregnancy-specific conditions under investigation.

## Results

As of 2021 (at the time of the study), we identified a total of 444 candidates, most of which were for preterm labour or birth (178 candidates), followed by pre-eclampsia or eclampsia (153 candidates). Fewer candidates were found for intrauterine growth restriction (63 candidates), postpartum haemorrhage (39 candidates), and only 11 candidates were found for intrapartum fetal distress. Within the 444 candidates, there were 68 candidates that were investigated for more than one condition, thus appearing more than once in the total count. Overall, there were 344 unique candidates contained within the dataset.

In total, 224 candidates (50%) were in active development at the pre-clinical or clinical stage, with 220 candidates (50%) inactive. More candidates were inactive than active for preterm labour or birth (110 [62%] of 178) and fetal distress (10 [91%] of 11), whereas for pre-eclampsia (90 [59%] of 153 candidates) and intrauterine growth restriction (38 [60%] of 63 candidates) more candidates were in active development than inactive.

Two-thirds of candidates for all conditions were repurposed (291 [66%] candidates; figure A). The predominance of repurposed candidates was more pronounced among active candidates (166 [74%] of 224 candidates; figure B). In total, 284 (64%) of the 444 candidates were drugs, 59 (13%) were biological products, and 101 (23%) were dietary supplements (figure C). Within the drugs and biological products categories, a number of nanoparticle-based candidates were under development for all conditions: eight for preterm labour or birth, five for intrauterine growth restriction, two for pre-eclampsia or eclampsia, one for postpartum haemorrhage, and one for fetal distress. Some of these were novel, nanotechnology-based deliveries of an existing medicine—for example, nanoparticle delivery of nifedipine for preterm birth or labour.

**Figure:**
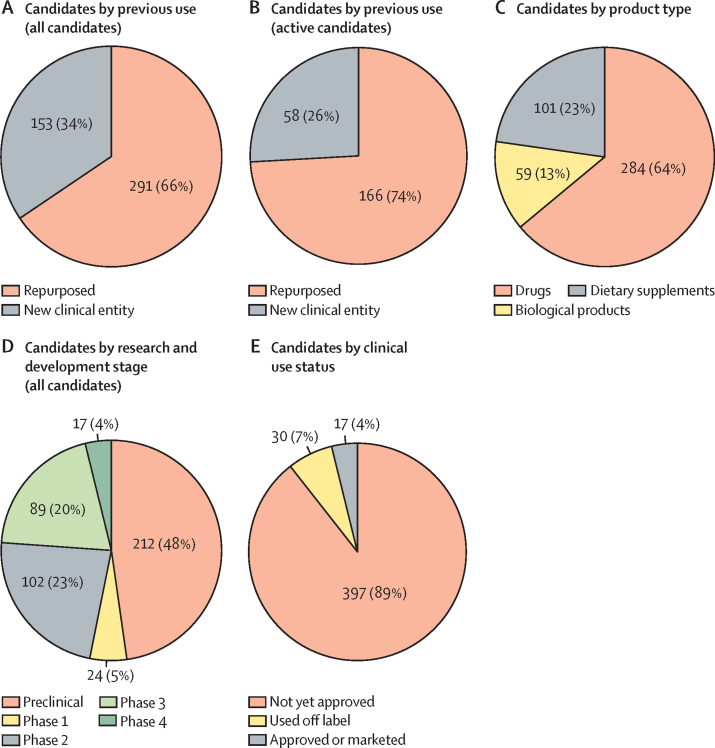
Overview of the number of maternal health medicine candidates by previous use (A, B), product type (C), research and development stage (D), and clinical use status (E)

We identified many preclinical candidates (212 [48%] candidates; figure D). Most preclinical candidates were for preterm birth or labour (107 candidates; 50% of all preclinical candidates and 24% of all candidates). There were 24 candidates in phase 1 (5% of the entire database), 102 (23%) in phase 2, 89 (20%) in phase 3, and 17 (4%) in phase 4. These R&D stage proportions were similar when considering candidates for preterm birth or labour, pre-eclampsia or eclampsia, and intrauterine growth restriction. The stage with the largest proportion of candidates was preclinical development, a small proportion were in phase 1, and there was a relatively even split for phase 2 and phase 3. In contrast, postpartum haemorrhage had few preclinical and phase 1 candidates, and a greater number of candidates in mid-stage and late-stage clinical development compared with preterm birth or labour, pre-eclampsia or eclampsia, and intrauterine growth restriction. Postpartum haemorrhage also had the largest number of approved candidates in phase 4 postmarketing or surveillance studies, compared with the other conditions. Fetal distress was an outlier with very few candidates overall. All fetal distress candidates were in phase 2, except for a haemoglobin vesicle via nanoparticle delivery, which was at the preclinical stage. This product was also under investigation for pre-eclampsia or eclampsia and intrauterine growth restriction.

Within the 2000–21 period, only 17 medicines (4%) were specifically approved for use across the five conditions (figure E; appendix p 2). We identified 30 medicines (7%) that have evidence of being utilised off label in clinical practice, the majority of which are therapeutic medicines for pre-eclampsia or eclampsia, and preterm labour or birth. For example, we identified several antihypertensives that are routinely used off label to manage pre-eclamptic hypertension, as well as tocolytic drugs used to prolong gestation in women with preterm labour (appendix pp 3–4). The remaining 397 candidates (90%) were not yet approved for clinical use, nor were they routinely used off label. Of these investigational candidates, more than half were in early discovery or preclinical phases (211 [53%] of all not-yet-approved medicines). Six of the 17 approved and marketed medicines, and nine of the 30 medicines that were used off label, are currently approved for one of the five specific pregnancy-related conditions or are recommended by WHO for prevention or treatment (table; appendix pp 2–4).

**Table:** WHO-recommended medicines for pregnancy-specific conditions, their product development history, and approval status

		Archetype (purposely developed*or repurposed)	**Clinical use status for pregnancy-specific condition (off-label or approved)**
**Postpartum haemorrhage**^26^, ^27^, ^28^			
Oxytocin		Purposely developed for postpartum haemorrhage	Approved by SRAs for postpartum haemorrhage
Ergometrine or methylergometrine		Purposely developed for postpartum haemorrhage	Approved by SRAs for postpartum haemorrhage
Oxytocin plus ergometrine; fixed-dose combination (eg, Syntometrine)		Purposely developed for postpartum haemorrhage	Approved by SRAs for postpartum haemorrhage
Misoprostol		Repurposed	Off-label
Tranexamic acid		Purposely developed as antifibrinolytic agent, with the intention to be used for postpartum haemorrhage†^29^	Approved by SRAs for haemorrhage
Carbetocin		Purposely developed for postpartum haemorrhage	Approved by SRAs for postpartum haemorrhage; not approved by FDA
Heat-stable carbetocin		Purposely developed for postpartum haemorrhage	Approved by EMA (April, 2015); approved by Swissmedic under the Marketing Authorisation for Global Health Products procedure (May, 2020)
**Pre-eclampsia or eclampsia**^31^, ^32^, ^33^			
Low-dose aspirin^34^		Repurposed	Off-label
Antihypertensive drugs^35^, ^36^			
	Labetalol	Purposely developed as antihypertensive but not specifically developed for use in pregnancy and lactation†	Off-label
	Nifedipine	Purposely developed as antihypertensive but not specifically developed for use in pregnancy and lactation†	Off-label
	Methyldopa	Purposely developed as antihypertensive but not specifically developed for use in pregnancy and lactation†	Off-label
	Hydralazine	Purposely developed as antihypertensive but not specifically developed for use in pregnancy and lactation†	Off-label
Magnesium sulfate		Repurposed	Approved by SRAs for pre-eclampsia or eclampsia
Calcium supplement		Repurposed	Off-label
**Preterm labour or birth**^37^, ^38^			
Nifedipine		Repurposed; first reported in 1980 in an observational study to be an effective tocolytic agent^39^	Off-label
Atosiban		Purposely developed for preterm birth	Approved by EMA and in India and many other countries; not approved by FDA
Glyceryl trinitrate^40^		Repurposed	Off-label
Antenatal corticosteroids^41^			
	Betamethasone	Repurposed	Off-label
	Dexamethasome	Repurposed	Off-label
**Fetal distress**‡			
None		NA	NA
**Intrauterine growth restriction**‡			
None		NA	NA

FDA=US Food and Drug Administration. SRA=stringent regulatory authority.

* Purposely developed refers to a new chemical entity to specifically address the named use at the time of development.

† Referred to as repurposed in the analysis of this paper and in the online maternal health pipeline database.

‡ No WHO recommendations available.

More detailed information about the candidates for postpartum haemorrhage, pre-eclampsia and eclampsia, preterm birth or labour and intrauterine growth restriction can be found in detailed analyses of the database.^22^, ^23^, ^24^, ^25^

## Discussion

Although our view of the historical, maternal health R&D landscape is more detailed and appears to be larger (driven to a considerable degree by the expanded scope of this study) than in Fisk and Atun's study,^2^ the message remains the same: 15 years later there is still a dearth of innovation in medicines for maternal health. The market failures that have inhibited R&D investments for medicines designed for use during pregnancy remain unresolved.

Since the 1950s, only two drugs have been developed and registered specifically for pregnancy-specific conditions: atosiban and carbetocin. Atosiban, a tocolytic agent, has been on the market since the 2000s.^42^ Carbetocin is an oxytocic agent used to prevent excessive bleeding after childbirth or caesarean section and has been approved since 1997.^43^ Heat-stable carbetocin can be regarded as an innovation that increased the use case for carbetocin, although it is not technically a newly developed drug. Although many drugs are recommended and commonly prescribed for pregnancy-specific conditions—including nifedipine for preterm labour and misoprostol for postpartum haemorrhage—they remain off-label.^26^, ^37^ Misoprostol, for example, was initially developed and approved for the treatment of stomach and duodenal ulcers in individuals taking nonsteroidal anti-inflammatory drugs.^44^

With the knowledge that Fisk and Atun only reported 67 candidates in 2008, identifying 444 candidates suggests, at first glance, substantial growth in the maternal health medicines pipeline. However, our methodology was broader, including candidates that were not in active development (50%) and those that were in active development but repurposed (37%). As the pipeline covers an R&D period of more than 20 years, the result could also give the impression that there are many new drugs that will soon be available for use. However, of the 17 products identified as approved or marketed, some have been withdrawn (eg, injectable hydroxyprogesterone caproate [Makena]^3^), and others are not recommended by WHO due to their side-effects and unfavourable safety profiles (eg, carboprost, sulprostone, ritodrine, and other betamimetic drugs).^26^, ^45^

In the table, we summarise current WHO recommendations for three of the five pregnancy-specific conditions (there are no WHO recommendations on medicines specifically for prevention or treatment of intrauterine growth restriction and fetal distress) and have included candidate information from our maternal health pipeline database. There are two key takeaways.

First, of the 19 WHO-recommended medicines, only seven were purposely developed for any of the five pregnancy-specific conditions we studied, meaning that the remaining 12 medicines are repurposed or developed for non-pregnancy indications, such as hypertension. Although repurposing allows more drugs to be used during pregnancy, safety and efficacy during pregnancy should still be assessed in appropriately designed studies. We also found that some drugs were being used completely outside of their purposive development indication—eg, nifedipine, a blood pressure medication, to stop uterine contractions. Six of the purposefully developed medicines are used for the prevention or treatment, or both, of postpartum haemorrhage, and one medicine is used for the management of preterm birth. There are no medicines purposely developed for pre-eclampsia or eclampsia. Second, less than half of the recommended medicines (eight of 19) are approved for a specific pregnancy-related condition. The off-label use of these medicines does not necessarily mean their safety and efficacy has not been substantiated in clinical trials for these pregnancy-specific conditions, but the reason why they have not been approved remains in question. In short, there are few purposely developed maternal health medicines, and the majority of WHO recommended medicines are not approved for pregnancy-specific conditions.

Repurposed drug research remains the dominant pattern in the maternal health R&D pipeline. Although exploring new indications for those candidates that are already marketed and proven safe in pregnancy is an attractive, lower cost (and more pragmatic) option when compared with newly developing drugs, it highlights the structural challenges to advancing new chemical or biological entities for obstetric conditions more broadly. Among the 78 candidates that are currently active and are in phase 3 or phase 4 trials, only ten are new chemical or biological entities.

This comprehensive pipeline analysis has some limitations. In developing this database, our aim was to keep the focus on medicines being developed or used specifically for prevention or treatment of these five pregnancy-specific conditions. However, as described in the methods section, the pipeline does not include classes of medicines (such as labour induction agents or antibiotics) that have more generalised or multiple applications in pregnant women. These drugs might still be used as part of the prevention or management of the conditions of interest. For example, labour induction drugs can be used to treat pre-eclampsia, or antibiotics provided after premature rupture of membranes.

### Main barriers to maternal health drug innovations

#### Market challenges

The so-called drug drought in maternal health described by Fisk and Atun^2^ 15 years ago is continuing, with the same market challenges present today. One major factor is the unwillingness of pharmaceutical companies to invest, develop, and test drugs in and for pregnant individuals.^13^ Bringing a new medicine to market for a pregnancy condition is challenging. The thalidomide and diethylstilbestrol tragedies brought a risk-averse culture and strict, inflexible regulatory oversight. What was not considered was that both tragedies could have been averted or had a smaller adverse effect if those drugs had gone through rigorous trials. Even today, the reproductive toxicity or teratogenic potential of many medicines is not known. The unknown magnitude of risk leads insurers to act cautiously, resulting in higher trial and liability costs for pregnancy medicines, and pharmaceutical companies are (understandably) concerned with reputational risk and potentially substantial damages.

Clark and colleagues estimated that to bring a novel maternal health medicine to the market, a pharmaceutical company would need to invest an additional US$5·7 million compared with the overall cost for other therapeutic areas, with around $950 000 being spent on pharmacokinetic studies and $4·7 million being spent on safety and efficacy studies.^46^ The pharmaceutical market is also quite asymmetrical. Six markets (USA, Japan, Germany, France, UK, and Canada) accounted for three-fifths of the revenue generated by pharmaceutical companies in 2011.^47^ Accordingly, pharmaceutical companies prefer to register their heavily invested, patented drugs in high-income countries to maximise revenue opportunities before introducing them in LMICs, viewing large investments in maternal health as high risk and low profit in comparison.

#### Gender bias

The development of technologies and solutions to myriad issues have, in general, systematically and persistently ignored women, who represent half of the global population. Such biases range from seat belts in cars to medicines that are developed on the basis of male physiology and characteristics.^48^ Pregnant women were excluded from COVID-19 trials and then advised to receive a vaccine, even when the evidence base included very few pregnant women.^49^, ^50^ Although female individuals metabolise drugs differently to male individuals, with implications for treatment regimens, these are rarely accounted for in clinical trial design or in approved medicines dosing.^51^

#### Recruitment of pregnant and lactating individuals into clinical trials

Recruitment to intervention trials is a challenge for pregnant individuals, their families, care providers, and researchers. The norm has been to exclude people who are pregnant or lactating from drug and vaccine trials.^52^ However, there are many other areas in health care in which trial recruitment is challenging for different reasons (eg, in unconscious patients, newborn babies, or children), but these challenges have not stopped research in these areas. Indeed, people who are pregnant have to consider possible risks to both themselves and their babies, often without the evidence needed to make a fully informed choice. Care providers might have difficulty themselves in requesting participation from pregnant individuals and their partners or families. The fact that many people take medications during pregnancy and lactation suggests that accurate and appropriate communication, and information sharing about risk and potential benefits to others (altruistic objective), could be helpful in increasing research in this population.^53^

### Solutions and future directions

Although we can state with reasonable certainty that there will be no game changing drug emerging for pre-eclampsia, intrauterine growth restriction, or preterm labour before the end of the 2030 Sustainable Development Goals period, there are also reasons to be optimistic. Engagement and interest to accelerate innovation for pregnant individuals is growing, and new initiatives have formed. There are challenges, but solutions are available that will not only apply for pregnancy-specific conditions, but any condition that negatively affects pregnancy. It is encouraging to see initiatives such as Malaria in Mothers and Babies from Medicines for Malaria Venture,^54^ the Innovative Medicines Initiative-funded ConcePTION project, and the recently published Women's Health Innovation Opportunity Map^55^ as a collaborative framework to advance women's health innovation.

#### Regulatory agencies

The main gatekeepers of R&D acceleration—the European, USA, and UK stringent regulatory authorities—have made substantial progress in incentivising R&D for pregnancy medicines. These agencies are open to further developments, with the onus on researchers and other stakeholders to propose and drive forward interest in new solutions.^13^, ^56^, ^57^ One recommendation under consideration in the UK is introducing (or at least piloting) a maternal investigation plan as a new licensing requirement for medicines, similar to the paediatric investigation plan, which is widely seen as a successful model that accelerated drug development for children.^57^ Physiology-based pharmacokinetics modelling and upscaled deployment of artificial intelligence are other areas in which development phases could be shortened or accelerated.^54^ Research groups could take a more proactive role in supporting regulatory agencies to leverage and implement these types of changes.

#### Insurance de-risking

Insurance de-risking is difficult, although there are some potential solutions. One possible innovation could be to develop a catastrophe risk fund or captive insurance scheme in which multiple stakeholders pool resources to cover the costs of R&D-related insurance and liability.^58^ Insurance de-risking would require partnerships and financial commitments between insurers, donors, and developers. Other initiatives, such as advance market commitments, have been used in vaccine development.^59^ Typically offered by a government or donor, an advance market commitment is a contract that guarantees a viable initial market (sale price and manufacturing capacity) for a product once it is successfully developed. Most often, advance market commitments are used in settings where the cost of developing a new product is too high to be worthwhile for the private sector without a guarantee of a certain quantity of purchases in advance.^28^ A result of this commitment—for which feasibility depends heavily on matching the target product profile and reasonable purchase prices—is the acceleration of development and introduction of new drug candidates in LMICs as market risks have been removed. The mitigation of financial and reputational liability risks, increased investment through cost-sharing, and improving maternal health supply channels to support innovation uptake and pricing considerations for LMICs are solutions that are essential for pharmaceutical industry engagement and investment.^60^

#### Product partnerships

It is likely that no single entity can solve the problem of sparse R&D or new drugs for pregnancy-specific conditions. Many innovations are initiated by academia or start-up companies, or both, and although they can patent their innovations and secure ownership, their chances of bringing a product into clinical use in places where the need is greatest is close to zero under current market conditions. These innovators need either industry partners or large philanthropic donors to support them or to commit to purchasing their products (in the case of industry). Obtaining support or partnerships from donors and industry partners is challenging because it requires a long-term commitment (10–15 years) both financially and strategically. Partnerships that include funding commitments, development support, market assessment, and strategising by different entities are likely to be more successful.

#### Advocacy

Although the exclusion of pregnant women's participation in clinical trials of COVID-19 medicines and vaccines proved newsworthy, it does not mean that medicines and vaccines for use in pregnancy will be prioritised in the future. We need collective action, including ownership from governments of countries where the burden is highest, to make inclusion of pregnant participants in clinical trials the norm rather than the exception. Importantly, we must engage civil society organisations to underscore the urgency of the matter and create demand from pregnant people, their families, and health-care professionals to make real progress in this field. In 2018, the FDA published a guideline document with considerations to include pregnant women in clinical trials.^61^ In the same year, the Task Force on Research Specific to Pregnant Women and Lactating Women published a list of 15 recommendations with the central theme for the need to “alter cultural assumptions that have significantly limited scientific knowledge of therapeutic safety, effectiveness, and dosing for pregnant and lactating women”.^62^ The recommendations include among others to increase the quantity, quality, and timeliness of research, implementation of a liability mitigation strategy for conducting research and evaluating new therapeutic products, and to create a public awareness campaign to engage the public and health-care providers in research on pregnant women and lactating women.^62^ These recommendations are echoed in other frameworks discussing ethical guidance for research involving pregnant women.^63^, ^64^ A strong recommendation shared by many is the reclassification of pregnant women from being considered a vulnerable population to a scientifically complex population, and changing the presumption of exclusion to one of inclusion.^53^, ^65^

## Conclusion

We are living in an era with unprecedented scientific and technological developments, including precision medicine, artificial intelligence-assisted drug development, and digital solutions that have revolutionised care in many disease areas. With interest and enhanced engagement with pharmaceutical companies, regulatory agencies, national research institutions, and civil society organisations, we are confident that positive change is possible for the development of maternal health medicines too.

### Contributors

Conceptualisation: All authors. Data collection and maternal health pipeline curation: AT and MG. Formal analysis: AT, MG, AA, ARAM, JPV, and AMG. Funding acquisition: AMG, LC, and AA. Methodology: AT, MG, AA, ARAM, JPV, and AMG. Project administration: AA. Supervision: JV and AMG. Drafting the figures: AA. Writing of the original draft: AA. Review and editing: all authors. All authors had access to all the data in the study and had final responsibility for the decision to submit for publication. AMG, JPV, ARAM, AA, AT, and MGAA have accessed and verified the data.

## Declaration of interests

We declare no competing interests.
