# Supplementary material for: The drug drought in maternal health: an ongoing predicament
Source: Lancet Glob Health. 2024 Jun 12;12(7):e1174–83. doi: 10.1016/S2214-109X(24)00144-X (PMC11194164; doi:10.1016/S2214-109X(24)00144-X)
Supplement: Supplementary appendix [file mmc1.pdf]

THE LANCET
Global Health

Supplementary appendix

This appendix formed part of the original submission and has been peer reviewed. We post it as supplied by the authors.

Supplement to: Ammerdorffer A, McDougall ARA, Tuttle A, et al. The drug drought in maternal health: an ongoing predicament. *Lancet Glob Health* 2024; **12:** e1174–83.

Table of contents

1. *Supplement Table 1.*
   Candidates approved and marketed for use across the five pregnancy conditions in the period 2000-2021.

2. *Supplement Table 2.*
   Candidates used off label across the five pregnancy conditions in the period 2000-2021.

Supplement Table 1. Candidates approved and marketed for use across the five pregnancy conditions in the period 2000-2021.

Condition*	Candidate
**Postpartum haemorrhage**	Carbetocin**
	Carboprost
	Dinoprost
	Ergometrine/methylergometrine **
	Fresh frozen plasma (including cryoprecipitate)
	Oxytocin **
	Oxytocin plus ergometrine in a fixed dose combination (i.e. syntometrine) **
	Prothrombin complex concentrate
	Sulprostone
**Pre-eclampsia/ eclampsia**	Magnesium sulphate **
**Preterm labor/birth**	Allylestrenol
	Atosiban**
	Fenoterol
	Hexoprenaline
	Injectable 17-alpha-hydroxyprogesterone caproate***
	Isoxsuprine
	Ritodrine

* No candidates are approved and marketed for foetal distress and intrauterine growth restriction
** Candidate currently still approved/marketed and/or recommended by WHO.
*** Withdrawn from the market in 2023

Supplement Table 2. Candidates used off label across the five pregnancy conditions in the period 2000-2021.

Condition	Pharmacological subgroup	Candidate	Marketed for
**Postpartum haemorrhage**	Vasopressin and analogues	Desmopressin	Haemophilia A; Von Willebrand's Disease; Diabetes insipidus; Nocturnal enuresis
	Herbs	Herb – Shenghua decoction*	Dietary supplement; Uterine bleeding
	Prostaglandins	Misoprostol**	Gastric ulcers; Termination of pregnancy
**Pre-eclampsia/ eclampsia**	Beta-blocker	Atenolol*<br>Labetalol**<br>Metoprolol	Hypertension; Cerebrovascular accident; Cardiovascular disease; Atherosclerosis; Angina pectoris; Heart failure; Myocardial infarction; Arrhythmia
	ACE inhibitor	Captopril*<br>Enalapril	Heart failure; Hypertension; Left ventricular dysfunction
	Mineral	Calcium**	Dietary supplement
	NSAIDs	Aspirin**	Pain; Inflammation; Fever
	Imidazoline receptor agonists	Clonidine*	Hypertension
	Sulfonamides	Furosemide	Congestive heart failure; Oedema; High blood pressure
	Central alpha-2 adrenergic agonists	Methyldopa**	Hypertension
	Calcium channel blockers	Nifedipine**	Hypertension
	Antiepileptics	Phenytoin*	Generalized tonic-clonic status epilepticus; Seizures during epilepsy
	Nitroferricyanide derivatives	Sodium nitroprusside	Severe hypertension; Acute heart failure
**Preterm labor/birth**	Corticosteroids	Betamethasone**<br>Dexamethasone**	Inflammatory diseases; Skin conditions; Autoimmune disorders; Allergies; Asthma
	Calcium channel blockers	Nicardipine*<br>Nifedipine**	Hypertension; Stable angina
	NSAIDs	Indomethacin	Acute pain
	Osmotically acting laxatives	Magnesium sulphate **	Pre-eclampsia/eclampsia (seizures); Magnesium deficiency; Cardiac arrhythmia; Soaking minor cuts or bruises
	Beta-adrenoreceptor agonists	Orciprenaline*<br>Salbutamol*<br>Terbutaline	Bronchospasm; Asthma; Chronic obstructive pulmonary disease
	Progestins	Progesterone, natural, micronized (vaginal/topical)	Menopausal hormone therapy; Secondary amenorrhea; Gynaecological disorders; Fertility
	Acetic acid derivatives	Sulindac*	Inflammation; Pain

**Fetal distress**	Selective beta-2-adrenoreceptor agonists	Salbutamol*<br>Terbutaline*	Asthma; Chronic obstructive pulmonary disease
**Intrauterine growth restriction**	Estren derivatives	Allylestrenol*	Recurrent or threatened miscarriage; Preterm labour/birth

* Candidate currently not in active development for this pregnancy condition

** Candidate currently recommended by WHO
